# Expelling Stress for Primary School Teachers: Self-Affirmation Increases Positive Emotions in Teaching and Emotion Reappraisal

**DOI:** 10.3390/ijerph13050500

**Published:** 2016-05-13

**Authors:** James Morgan, Lisa Atkin

**Affiliations:** Psychology Group, Leeds Beckett University, Leeds LS1 3HE, UK; lisaatkin@hotmail.co.uk

**Keywords:** teaching, stress, anxiety, well-being, self-affirmation, emotions in teaching, emotion regulation, coping

## Abstract

The aim of the present pilot study was to assess the effect of a brief work-related self-affirming implementation intention (WS-AII) on the well-being of primary school teachers. Participants were randomly allocated to one of two conditions: one in which they were asked to create a WS-AII or one in which they were asked to create a control implementation intention (C-II). State anxiety was measured pre- and post-manipulation, self-efficacy at post-manipulation only, and emotions in teaching and emotion regulation at baseline and at a two-week follow-up. There were statistically significant differences between the WS-AII condition and the control. Teachers who created work-related self-affirming implementation intentions reported an immediate reduction in state anxiety. Positive effects extended over the two-week period, with teachers in the WS-AII condition also reporting more positive emotions in teaching and the use of reappraisal emotion regulation strategies rather than emotion suppression. Results suggest that the integration of the WS-AII into existing organisational practice may be of benefit to the well-being of teachers and other highly stressed workers.

## 1. Introduction

According to the UK Health and Safety Executive, teaching is a highly stressful job. In their survey of twenty-seven occupations, teaching was associated with the largest proportion of reported “high stress” [1]. Subsequent findings suggest that this situation has not changed. Johnson *et al.* [2] found teaching to be one of the six most stressful careers, ranking 2nd (out of 26) for both physical and psychological stress. The same study revealed that teachers also rate their levels of job satisfaction as lower than average. Absenteeism within education is also higher than in other sectors. In 2013, approximately 10.2 days per person were lost through sickness compared to 7.6 days in other industries [3]. Attrition figures for primary and secondary teachers show that 66% of teachers leave for reasons other than retirement and 40% leave within the first five years [3]. 

It is likely that role-specific factors rather than the school environment cause high stress. Teaching assistants and head teachers generally report very low levels of physical and psychological stress [4]. Emotional labour may be a contributory factor as teachers are more likely to have to deal with difficult class situations in which they must retain personal control in the face of challenging pupil behaviour [4]. In addition to emotional labour, the pressures of planning, teaching observations, and meeting pupil achievement targets are also likely to increase stress [4]. 

There is a need for interventions designed to reduce stress and improve the well-being of teachers. By enabling teachers to cope better, these interventions may also help to reduce stress-related sickness and improve the retention of teaching staff in mainstream compulsory education. The principal aim of the present pilot study is to test the effects of a brief work-related self-affirming implementation intention on immediate affect (state anxiety), longer-term role-specific workplace well-being (emotions in teaching), and emotion regulation (coping strategies). 

### 1.1. Self Affirmation Theory and Stress Reduction

According to self-affirmation theory [5], people are motivated to preserve a positive, moral, and adaptive self-image and to thereby maintain “self-integrity”. Thus, threats to the self elicit defensive information processing. According to Steele’s [5] self-affirmation theory, however, because people are motivated to defend their *global* sense of self-worth, self-affirmation in one domain (e.g., by recalling past acts of kindness) should reduce the need to be defensive when threatened in another domain (e.g., by job stress). In other words, if a person’s self-image can be “affirmed” in a domain that is important to them, this should act as a buffer against threats to the self and therefore reduce the impact on both physiological and psychological responses (see [6] review). 

Evidence suggests that drawing on self-resources can reduce the physiological and psychological impact of both laboratory-induced (see [7,8]) and naturally occurring (see [9]) stressors. For example, one study [8] reported a significant negative correlation between perceived self-resources and cardiovascular reactivity during a laboratory stressor, while another study [7] found that self-affirmation attenuated cortisol response to the Trier Social Stress Task [10]. In addition to the physiological response, Creswell *et al.* [7] found that participants with higher dispositional self-resources (e.g., trait self-esteem and optimism) who affirmed their core values reported the least psychological stress. 

To our knowledge only one laboratory-based study [9] has examined the effects of an experimental self-affirmation manipulation on stress responses to an everyday stressor (academic stress prior to an exam). While there was a cumulative increase in epinephrine (an indicator of sympathetic nervous system activation) in the urine samples of undergraduates in the control condition in the run up to an exam, there was no concomitant change in the self-affirmation group. Outside of the laboratory, Morgan & Harris [11] extended the focus on everyday stressors to examine the effects of self-affirmation on the anxiety experienced by further education workers during a period of organisational downsizing. In response to criticisms of existing methods of self-affirming and the lack of experimentally equivalent controls, Morgan & Harris [11] utilised an adapted self-affirming implementation intention, resulting in both short-term and long-term anxiety reduction. 

### 1.2. Self Affirming Implementation Intentions

Experimental self-affirming manipulations have typically taken the form of value scales (e.g., [12]). From a list of statements describing different domains of self-worth, participants are encouraged to identify the values (e.g., aesthetic, social, *etc.*) that are most important to them. Whilst this method can be useful in inducing self-affirmation, it has been criticised because it is often difficult for researchers to provide core value statements that are salient for large and diverse participant populations [13]. As such, an alternative method involves asking participants to write at length about their chosen personal values. Again, whilst this method has been successful in studies with relatively small participant sample sizes [14,15], it has proved to be impractical for larger samples due to its time consuming nature. 

Napper, Harris, and Epton [13] argue that, due to the length and complexity of value-based methods, researchers have also found it consistently difficult to devise appropriately equivalent non-self-affirming control tasks in experimental self-affirmation studies. Additionally, problems arise when faced with participants with low literacy/education levels, as the task requires verbal fluency [13]. To address these latter concerns, Armitage, Harris, and Arden [16] developed and tested a briefer, standardised self-affirmation manipulation based on implementation intentions [17]. 

Implementation intentions are specific kinds of if-then plans that work by encouraging people to link in memory-critical situations with appropriate behavioural responses. Implementation intentions have been used with some success to change health behaviors [18]. The principal idea behind implementation intentions is that the salience of critical situations is enhanced when they are encountered in the environment and that appropriate behavioural responses are triggered automatically [17]. Research has shown that forming implementation intentions can have a significant impact on future behaviour over the longer term [18]. Based on the work of Harris, Napper, Griffin, Schuez, and Stride [19] (2011), Armitage *et al.* [16] sought to develop a brief manipulation in which participants were asked to form an implementation intention (an if-then plan) to self-affirm by simply writing out a sentence made up of a stem and a chosen response. For “the self-affirming implementation intention” (S-AII) participants were presented with the stem, adapted from Harris *et al.* [19], “*If I feel threatened or anxious, then I will …*” where “*feeling threatened or anxious*” is the critical situation, and a choice of appropriate self-affirming responses include: “*thinking about the things I value about myself*” and “*remembering things that I have succeeded in*” [19]. More recently, Morgan & Harris [11] adapted the S-AII to create the work-related self-affirming implementation intention (WS-AII) as well as a non-self-affirming implementation intention task as an active control in order to achieve greater “control equivalence” (see [13]). In a population of organisational downsize survivors from a UK further education college, feelings of anxiety and depression were measured before and after the intervention or control task, and three weeks later. Job satisfaction, self-efficacy, and self-esteem were also measured. There were statistically significant differences between the WS-AII condition and the control. Workers who created work-related self-affirming implementation intentions reported an immediate reduction in anxiety. This reduction was also observed in their appraisal of job-related anxiety three weeks later. There were no significant effects of WS-AII’s on depression, job satisfaction, or self-esteem. There was, however, a significant effect on self-efficacy, with workers in the WS-AII condition reporting greater self-efficacy.

### 1.3. Rationale for Present Study

The research carried out by Morgan & Harris [11] is the first to show beneficial effects of a brief self-affirming implementation intention on anxiety in further education college staff during organisational downsizing. However, as the authors suggest, further work is required in order to establish whether these benefits can be replicated in other work populations during everyday operations. Additionally, it is suggested that the specificity of the self-affirmation manipulation and outcome measures may be increased to improve the validity of findings in particular occupational settings. As such, the present pilot study will investigate whether a teaching-specific WS-AII can reduce the immediate stress response (state anxiety) of primary school teachers working in mainstream, compulsory education. The study will also evaluate the longer-term effect of the WS-AII on the domain-specific appraisal of positive (well-being) and negative (stress) emotions attributed to teaching over a two-week period. 

Although it is thought that self-affirmation effects may be mediated by increases in self-efficacy (e.g., [11,20]), the mechanics of self-affirmation remain unclear [21]. The present study is the first to also explore the effects of self-affirmation on emotion regulation processes (coping strategies) over time (a two-week period). Gross & John [22] found that strategies such as reappraisal and suppression have implications for emotional well-being. It is theorised that antecedent-focused reappraisal strategies alter subsequent emotional trajectory and are effective in downgrading negative emotions [22]. Suppression strategies, however, are response-focused, serving to modify behavioural responses to emotion rather than the emotion itself. This strategy is cognitively demanding and can cause incongruence between emotion and behaviour, which in turn has been linked to depressive symptoms [23]. Conversely, reappraisal strategies are associated with greater well-being, with reappraisers experiencing greater positive emotions and lesser negative emotions [22]. 

In line with Morgan & Harris [11], to test its potential mediating effect, self-efficacy was also measured immediately post-manipulation.

Based on our review of previous literature and the rationale above, we propose the following four hypotheses: 

*Hypothesis 1*: There will be a significant immediate reduction in state anxiety in the WS-AII condition compared to the control condition.

*Hypothesis 2*: Teachers completing the WS-AII will report significantly greater levels of self-efficacy post-manipulation compared with those completing the C-II.

*Hypothesis 3*: Teachers completing the WS-AII will report significantly greater levels of positive emotions related to teaching and lower levels of negative emotions related to teaching at the two-week follow-up compared with those completing the C-II.

*Hypothesis 4*: Teachers completing the WS-AII will report a significant increase in the use of reappraisal strategies and a reduction in the use of suppression strategies at the two-week follow-up compared with those completing the C-II.

## 2. Method 

### 2.1. Participants and Design

Participants were teachers working in state primary schools in the north of England. We approached three semi-rural schools with a combined total teaching population of approximately 90 teachers. All three schools were classified as “Good” or better by the UK Office for Standards in Education, Children’s Services and Skills (Ofsted). Participants consisted of 14 men and 28 women (representing an approximate 47% response rate) aged between 23 and 52 (*M* = 33.04 years, *SD* = 8.13). Tenure ranged from 1 year to 21 years (*M* = 6.93 years, *SD* = 5.84). Eight participants identified themselves as being Newly Qualified Teachers (NQTs). NQTs are those that have gain qualified teacher status but as yet have not completed the statutory 12-month induction programme. NQTs are expected to complete this induction within five years of qualifying.

The research design was mixed. The between-participants variable was condition: Participants were randomised to either the work-related self-affirming implementation intention (WS-AII) condition or a control implementation intention (CII) condition. The within-participant variable was the time interval between baseline and post-manipulation follow-up (immediately after or two weeks later). There were six outcome variables. Emotions in teaching (with two subscales; positive/negative) and emotion regulation (with two subscales; reappraisal/suppression) were measured at baseline and at two weeks post-baseline. State anxiety was measured at baseline and immediately following the manipulation, and self-efficacy was measured post-manipulation only.

### 2.2. Procedure

Participants provided their informed consent before completing the study. The study was conducted in accordance with the Declaration of Helsinki, and the protocol was approved by the Local Ethics Committee at Leeds Beckett University (Project identification code: LVA/7129305/MSC). Prior to data collection, permission was sought from the head teachers of three small primary schools to approach teaching staff. Details of the study were then passed on to teachers by the head teacher via internal staff communication channels, informing them of the date on which the study would take place and emphasising that participation would be voluntary. The second author numbered all questionnaires before sorting them into a random order (using random number tables) and placing them in an unmarked folder. The front sheets of all questionnaires were identical so that the experimenter was blind to conditions.

On the date specified, the questionnaires were removed from the folder and one was placed in each of the staff pigeonholes, along with the consent forms, by an individual who was unaware of the conditions. A secure post box was positioned in the staff corridor in order for participants to deposit their questionnaires after completion. A similar procedure was used at follow-up, with the addition of a written thank you sent to all staff with a study debrief (in which all workers were offered the opportunity to complete the work-related self-affirming implementation intention). The questionnaire content is described below.

The first page of the questionnaire provided participants with details of their ethical rights including a request for their consent, as well as instructions for completing the measures. Demographic measures and measures of emotions in teaching, emotion regulation, and feelings of state anxiety followed. The self-affirmation manipulation or control task appeared on the next page, followed by a measure of self-efficacy. State anxiety was then measured once more. Participants in the experimental and control conditions received exactly the same questionnaire content with the exception of the self-affirmation or control task. 

### 2.3. Materials

#### 2.3.1. The Work-Related Self-Affirming Implementation Intention (WS-AII)

The work-related self-affirming implementation intention was an adapted version of the brief work-related self-affirming implementation intention developed by Morgan and Harris [11] in which participants are provided with an implementation intention prompt in the form of a sentence stem, “*If I feel threatened or anxious about work, then I will…*”*.* This is followed by four options: “*…think about the things I value about myself*”, “*…remember things that I have succeeded in*”, “*…think about what I stand for*”*,* and “*…think about things that are important to me*”*.* Participants are asked to write out the stem and their chosen option on three blank lines, with “*If…*” at the start of the first blank line. To reflect the specific teaching focus in the present study, the stem was adapted to read, “*If I feel threatened or anxious about teaching, then I will…*”*.*

#### 2.3.2. The Control Implementation Intention (CII)

The control implementation intention (CII) was developed by Morgan and Harris [11]. Akin to the work-related self-affirming implementation intention, to form the control implementation intention, participants were asked to rewrite the sentence stem followed by their chosen option on three blank lines, beginning with “*If…*”*.* Participants were asked to write out the same sentence stem as in the experimental condition. However, they were then required to choose one of four statements, which did not give participants the opportunity to self-affirm. These statements were adapted by Morgan and Harris [11] from existing control tasks. The first option, “*…think about the shops and buildings I pass on a journey I travel regularly*”, was taken from the journey control conceived by Napper *et al.* [13]. The second option, “*…remember the food I have eaten in the last 48 hours*”, was adapted from Cohen’s [24] food control, and the third and fourth options, “*…think about the most satisfying season of the year*”, and “*…think about the best flavour for ice-cream*”, were from the personal opinion survey [25]. 

### 2.4. Measures

#### 2.4.1. Pre-Manipulation

An adapted version of Trigwell’s [26] 20-item Emotions in Teaching Inventory (ETI) was used to measure domain-specific work stress. Participants were asked to provide their level of agreement with 10 statements measuring *positive emotions towards teaching* (motivation, pride, confidence, satisfaction, and happiness) and 10 statements measuring *negative emotions towards teaching* (anxiety, embarrassment, frustration, boredom, and annoyance). Responses were recorded on a five-point scale ranging from *strongly disagree* (1) to *strongly agree* (5). Because the original measure was intended for university lecturers in Australia the statements were adapted slightly to make them directly relevant to primary teachers in the UK, for example, “*I am motivated by my teaching role in this course*” was changed to “*I am motivated by my role as a teacher*”. Following the standard procedure for use of the scale, responses to the positive and negative items were aggregated separately for each participant to create two subscales. Cronbach’s alpha for each subscale indicated good internal reliability (positive emotions α = 0.93; negative emotions α = 0.86).

Gross & John’s [22] Emotion Regulation Questionnaire (ERQ) was used to measure how participants use the *emotional regulation* strategies of *suppression* and *reappraisal*. The ERQ contains 10 statements, each of which clearly indicate the emotional regulatory process it intends to measure, such as “*I control my emotions by changing the way I think about the situation I’m in*” (reappraisal) and “*I control my emotions by not expressing them*” (suppression). Participants were asked to rate each of the 10 statements on a scale of 1 (strongly disagree) to 7 (strongly agree). Participant responses were aggregated to create two subscales, one for suppression (α = 0.82) and one for reappraisal (α = 0.94).

*State anxiety* was measured using the state version of Marteau and Bekker’s [27] short form of the Spielberger State-Trait Anxiety Inventory [28]. Respondents were asked to indicate the extent to which they were experiencing six affective states at that present moment in time on four-point scales ranging from *not at all* (1) to *very much* (4). The six states are “*I feel calm*”,“*I am tense*”, “*I feel upset*”, “*I am relaxed*”, “*I feel content*”, and “*I am worried*”*.* After reverse-scoring the responses to the positively worded statements, high scores represent greater state anxiety (*α* = 0.84).

#### 2.4.2. Post-Manipulation

Schwarzer and Jerusalem’s [29] general self-efficacy scale was used to measure levels of *self-efficacy*. Participants were asked to rate their degree of agreement with 10 statements about their performance, such as “*If I am in trouble, I can usually think of a solution*” and “*I am confident that I could deal efficiently with unexpected events*”*.* Responses were made on four-point scales ranging from *not true at all* (1) to *exactly true* (4). High scores represent greater self-efficacy (*α* = 0.90).

To assess the immediate impact of the self-affirming manipulation on anxiousness, *state anxiety* was measured again, using the measure described above. Again, Cronbach’s alpha indicated good internal reliability (*α* = 0.88). 

#### 2.4.3. Two-Week Follow-Up

*ETI* and *ERQ* were measured again at the two-week follow-up in the same manner as pre-manipulation.

## 3. Results

### 3.1. Randomisation Checks

The effectiveness of randomisation was checked using multivariate analysis of variance (MANOVA). The independent variable was condition with two levels: WS-AII and C-II. The dependant variables were age, gender, tenure, baseline positive and negative ETI scores, ERQ (reappraisal and suppression), and state anxiety. The multivariate test, *F* (8, 33) = 0.42, *p* = 0.89 $\eta_{p}^{2}$ = 0.09, and all univariate tests, *Fs _univariate_*(1, 40) = 0.001 to 2.05, *ps* > 0.16, $\eta_{p}^{2}$s < 0.05, were not significant, which suggests that randomisation to condition was successful.

### 3.2. Effects on State Anxiety amd Self-Efficacy

The immediate effects of the manipulation were tested using multivariate analysis of covariance (MANCOVA); see descriptive statistics in Table 1) with condition as the independent variable (WS-AII *vs.* CII), self-efficacy, and post-manipulation state anxiety entered as the dependant variables, and pre-manipulation state anxiety (WS-AII *M* = 2.36, *SD* = 0.61; CII *M* = 2.20, *SD* = 0.63) entered as a covariate. The multivariate test, using Pillai’s Trace *V* = 0.38, *F* (2, 38) = 11.33, *p* < 0.001, $\eta_{p}^{2}$ = 0.38, (observed power = 0.98) was significant, as was one of the three univariate tests. There was a significant difference between conditions in state anxiety scores, *F* (1, 39) = 24.29, *p* < 0.001, $\eta_{p}^{2}$ = 0.38 (observed power = 0.99). There was no significant effect for self-efficacy scores, *F* (1, 39) = 0.25, *p* = 0.62, $\eta_{p}^{2}$ = 0.006. Self-affirmation was associated with lower post-manipulation state anxiety compared to the control. 

### 3.3. Effects on Emotions in Teaching, and Emotion Regulation 

A MANCOVA was performed with condition as the independent variable, follow-up positive and negative ETI scores as the dependant variables, and pre-manipulation positive (WS-AII: *M* = 3.46, *SD* = 0.84; CII: *M* = 3.64, *SD* = 0.73) and negative (WS-AII: *M* = 2.63, *SD* = 0.93; CII: *M* = 2.38, *SD* = 0.74) emotion scores as the covariates. The multivariate test, using Wilks’ Lambda—ᴧ = 0.82, *F* (2, 37) = 4.02, *p* = 0.03, $\eta_{p}^{2}$ = 0.18 (observed power = 0.68) was significant, as was one of the univariate tests. There were significant differences between groups in follow-up positive ETI scores, *F* (1, 38) = 8.24, *p* = 0.007, $\eta_{p}^{2}$ = 0.18 (observed power = 0.80), but not for follow-up negative ETI scores, *F* (1, 38) = 1.39, *p* = 0.25, $\eta_{p}^{2}$ = 0.04. Self-affirmation was associated with higher positive emotions towards teaching at follow-up compared to the control.

A MANCOVA was performed with condition as the independent variable, reappraisal, and suppression at follow-up entered as the dependant variables and pre-manipulation reappraisal (WS-AII: *M* = 4.41, *SD* = 1.22; CII: *M* = 4.90, *SD* = 0.96) and suppression (WS-AII: *M* = 3.80, *SD* = 1.42; CII: *M* = 3.40, *SD* = 1.24) as the covariates. The multivariate test, using Wilks’ Lambda—ᴧ = 0.62, *F* (2, 37) = 11.57, *p* < 0.001, $\eta_{p}^{2}$ = 0.39 (observed power = 0.99) was significant, as were both of the univariate tests. There were significant differences between groups in follow-up reappraisal scores, *F* (1, 38) = 18.32, *p* < 0.001, $\eta_{p}^{2}$ = 0.33 (observed power = 0.99), and follow-up suppression scores, *F* (1, 38) = 4.79, *p* = 0.04, $\eta_{p}^{2}\;$= 0.11, (observed power = 0.57). Self-affirmation was associated with lower suppression and higher reappraisal at follow-up compared to the control.

## 4. Discussion

The aim of the present pilot study was to assess the effects of a brief work-related self-affirming implementation intention on immediate affect (state anxiety), on role-specific workplace well-being/stress (positive/negative emotions in teaching) over a two week period, and on the emotion regulation strategies of primary school teachers (reappraisal/suppression). The potential mediating role of self-efficacy was also examined. Consistent with previous research, self-affirmation was associated with an immediate reduction in state anxiety (see [7,8,9,11]). Additionally, our findings support the notion that positive effects of WS-AII can persist for a number of weeks post-manipulation (see [11]). For the first time, self-affirmation was shown to increase domain-related positive emotions (towards teaching) and the use of more positive reappraisal coping strategies (as well as a concomitant reduction in emotion suppression) at the two-week follow-up. 

Despite these positive findings, there was no significant effect of the WS-AII on self-efficacy. This is not surprising given inconsistent results in relation to the relationship between self-affirmation and self-efficacy in previous studies [21]. The mechanics of self-affirmation are unclear. However, a recently published fMRI study [30] indicates that reflecting on personal values activates neural reward regions (*i.e.*, ventral striatum). The authors assert that this may be the first step towards locating the neural basis for self-affirmation. If this is the case, then a better understanding of the wide-ranging psychological and behavioural benefits may follow. 

The remainder of the discussion considers the theoretical and practical implications of our findings. 

### 4.1. Strenghts and Implications

The majority of research on self-affirmation and stress had been conducted in the laboratory [7,8,9,10]. Our applied study addressed the limited ecological validity evident in these studies by testing the immediate and short-term beneficial effects of a brief work-related self-affirming implementation intention in a sample of primary school teachers. As predicted, the WS-AII had a significant effect on positive emotions relating to teaching. Teachers who self-affirmed rated statements concerning motivation, pride, confidence, satisfaction, and happiness in teaching more highly at the two-week follow-up than did teachers in the control group (when controlling for baseline scores). Although general job-related anxiety was reduced for self-affirmed workers’ in a previous study (see [11]), which could also be cautiously interpreted as enhancing well-being, ours is the first study to clearly demonstrate role-specific well-being improvements over time. Somewhat surprisingly, although we utilised the more specific sub-scale measure of negative emotions in teaching, including anxiety, embarrassment, frustration, boredom, and annoyance, negativity was not significantly reduced for our self-affirmed teachers. It would seem sensible to predict that an increase in positive emotions in teaching would naturally lead to a change in negative emotions, particularly in light of previous findings concerning the use of reappraisal emotion regulation and the associated reduced frequency of negative feelings [22]. Our results can be explained in accordance with the findings of Steele [5] and Creswell *et al.* [7]. These authors assert that self-affirmation can cause people to view a threat more neutrally. Therefore, rather than diminishing their negative emotions, our self-affirmed participants were merely more accepting of them whilst feeling more positive about other aspects of teaching. 

An additional strength of the present study is that it is also the first to show that self-affirmation may lead to more positive coping strategies over time. After two weeks, teachers who had completed the WS-AII exhibited higher levels of emotion reappraisal and lower levels of emotion suppression. These findings are consistent with previous research espousing the longer-term benefits of implementation intentions for health behaviour change [18] and studies showing that participants are more responsive to health messages after self-affirming [14]. In both cases, it is probable that the reappraisal of emotions towards the behaviour in question has occurred, whereas the suppression of emotions seems unlikely. What is unclear in our study is the causal relationship between self-affirmation, emotion regulation, and positive emotions in teaching. This is perhaps an interesting avenue for future work.

Our results are in line with the applied work of Morgan and Harris [11] who were the first to test the use of the WSA-II in a work setting (downsize survivors at a further education college). Together, this study and ours strengthen the implication that the WS-AII may provide stress relief or heightened well-being for employees undertaking stressful job roles. The intervention was purposely designed to be brief in nature in order to minimise the impact of administration in the work place. Further research is needed in order to establish the best way to successfully integrate the WS-AII into existing work practices and to evaluate the efficacy of its use over a longer time period. For primary teachers it may be possible to disseminate information about the WS-AII and provide access to it at whole school level, such as during INSET (IN-SErvice Training day) or weekly staff meetings. Another option would be to integrate it with existing resources for combatting stress such as those provided by the various teaching unions and organisations such as the Teacher Support Network.

### 4.2. Limitations

Despite the positive implications associated with our findings, it is important to acknowledge potential limitations. First, we only followed workers over a relatively short period. Morgan and Harris [11] were restricted (by organisational pressures and practicalities) to a three-week follow-up, which was considered short. We faced similar challenges, commonly experienced in applied experimental research and thus our testing timeline was only two weeks. We recognise that it would be useful to see whether the significant positive effects can persist over a longer period. If practical, future studies should consider this, perhaps, as we suggest above, within the design of an evaluation study of intervention integration. A longitudinal approach would also help to overcome the limitations associated with the cross-sectional measurement of teaching-related well-being. Measuring the effects of self-affirmation at successive time points may provide a better evaluation of its efficacy in response to fluctuating levels of stress at particular times of the year (e.g., in the lead-up to examination periods). A longitudinal study may also afford the possibility of extending the measurement of well-being benefits to assess the longer-term impact of self-affirmation on sickness and attrition rates. 

Due to the applied research challenges mentioned previously, the present study recruited a relatively small sample of primary school teaching staff. Although this could be considered a limitation, and we would advise cautiousness in generalising our results beyond our specific UK-based primary school teacher population, the high observed power values suggest that statistical power was more than sufficient in our analyses. 

## 5. Conclusions 

Existent research findings suggest that affirming the self can reduce stress in laboratory conditions [7], amongst student populations [9], and, more recently, within the workplace [11]. The present study adds to these findings by showing that immediate anxiety can be reduced after self-affirming and that emotions in teaching and emotion regulation can be improved over time via the administration of a brief work-related self-affirming implementation intention. Restoring one’s global self-worth in this way may be an effective method to reduce stress and/or improve the well-being of those in the teaching profession, and in other highly stressed roles outside of teaching.

## Figures and Tables

**Table 1:** Comparison of experimental and control groups immediately post-manipulation and at the two-week follow-up.

Dependent Variables	Control, *n* = 21		Experimental, *n = 21*		*F* ^a^
	*M*	*SD*	*M*	*SD*	
Self-Efficacy ^b^	2.90	0.47	2.78	0.42	0.81
State Anxiety ^b^	2.22	0.64	1.65	0.35	24.29 ***
ETI Positive ^c^	3.62	0.71	3.71	0.83	7.98 **
ETI Negative ^c^	2.38	0.69	2.38	0.80	1.09
ERQ Reappraisal ^c^	4.80	0.90	5.13	0.88	16.89 ***
ERQ Suppression ^c^	3.32	1.25	3.20	1.15	3.29 *

^a^ Univariate *F*s testing post-manipulation/follow-up differences between control and experimental conditions, controlling for baseline scores: *df* = 1, 40; ^b^ Measures taken immediately post-manipulation; ^c^ Measures taken at the two-week follow-up. * *p* < 0.05; ** *p* < 0.01; *** *p* < 0.001.
